# Enhancing innovation speed through trust: a case study on reframing employee defensive routines

**DOI:** 10.1186/s13731-020-00143-3

**Published:** 2021-01-21

**Authors:** Christina Marie Mitcheltree

**Affiliations:** Department of Mechanical and Industrial Engineering, The Norwegian University of Science and Technology, 7491 Trondheim, Norway

**Keywords:** Trust, Innovation speed, Innovation adoption, Organizational innovation, Defensive routines, Health care, Case study

## Abstract

Trust in organizations plays an essential role for efficient innovation implementation. However, trust between managers and employees is under-communicated in relation to innovation speed. Innovation speed is related to innovation adoption, concerning new ways of performing laboratory services within the health sector. The purpose of this case study is to investigate how trust mechanisms may enhance innovation speed by reducing employee decisions to perform defensive routines. The focus is related to trust as a social condition for enhancing innovation acceptance in the context of management and organizing styles subject to the Norwegian Work Life Model.

The study found that a lack of employee participation and involvement may result in emotional tension, a sense of uncertainty, disconnect, and various defensive mechanisms towards management and the innovation. Consequently, employees’ attention, loyalty, and responsibility might be redirected away from the innovation.

## Introduction

Organizational innovation and change are significant for hospitals to maintain and enhance the quality of the health service offer at their laboratories. However, innovation adoption relies on managers’ ability to generate trusting relationships with their employees. This derives from the notion that being involved and considered in innovation decisions may limit defensive reactions to new ways of performing laboratory service tasks. Although this may be true, multi-location organizations with complex organizational structures may make dialogue between managers and employees more difficult to achieve. In the light of this, the paper emphasizes various trust mechanisms, and their ability to reduce defensive reasoning and strategies in relation to innovation implementation in complex organizations. The paper is written within the context of the Norwegian Work Life Model. Consequently, we emphasize key elements that may enhance the pace of innovation adoption within this context.

For innovation purposes, trust is stated as “an expectancy of reasonable and positive reactions by others in response to individual innovation attempts” (Clegg, Unsworth, Epitropaki, & Parker, 2002). Hence, as innovation involves risk and effort, innovation engagement may result either from an expectation of a positive response, from believing that suggestions will be heard, or from acquiring innovation benefits (Clegg et al., 2002). However, due to disciplinary differences, there is no collective confirmed operationalization of trust (Clegg et al., 2002).

Individuals naturally resist change (Lynn & Seth, 2008). Moreover, the speed at which an organization adopts an innovation relies on innovation characteristics and contextual factors (Webb & Pettigrew, 1999). The context thus depends on individual characteristics, the nature of the industry, stage, and type of innovation. Nevertheless, an institutional perspective of adoption is argued to be socially deterministic and involves managerial action (e.g., quality of leadership), human resources, and skills (Webb & Pettigrew, 1999). An underutilization of knowledge or ideas from, for example, employees of lower rungs of the hierarchy in the innovation elaboration process (e.g., participation) may thus act as a barrier to organizational value creation (e.g., organizational products and processes) (Yang & Konrad, 2011). Therefore, organizational defensive reasoning and defensive strategies involve avoidance, preventing organizational learning and capability (Argyris, 1986). Accordingly, it may be a barrier to change (in this case innovation speed) (Riley, Cudney, & Long, 2013). Since negative emotions should be avoided, there is a need for answers to effective ways that facilitate trust, caring, and commitment in organizations (Argyris, 2004). Moreover, what processes facilitate innovation adoption, and what characterizes innovative organizations, has not been answered properly (Damanpour & Schneider, 2006).

For this reason, the purpose of this paper is to examine the concept of emotion (main emphasis on emotional tension), defensive routines, and trust to understand *how trust may impact innovation speed*. A focus is placed on how trust may provide valuable and enhanced insight for multi-location organizations within complex organizational structures facing organizational innovation and change. The paper is based on a case study related to a hospital and its laboratory service. It is the result of an investigation done during a 3-month placement at the hospital to *seek understanding* of workers’ experiences with change and organizational innovation. Addressing innovation speed is related to understanding barriers to innovation, in this case emotional tension and defensive behavior, and how trust mechanisms on behalf of the laboratory employees may enhance innovation adoption in this context.

**Fig. 1:**
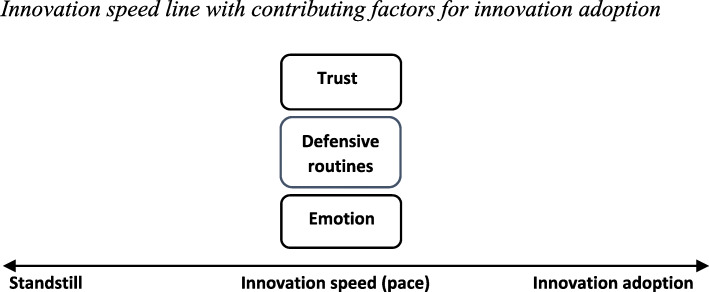
Innovation speed line with contributing factors for innovation adoption

To facilitate understanding of the innovation situation, the paper starts with an explanation of the paper context. The paper does not go into depth on the Norwegian Work Life Model but seeks to gain an *understanding* of the way the hospital has organized the innovation and the consequences for employees. Second, to be able to recognize the pace of employee innovation adoption within the hospital division, the concept of organizational innovation, innovation adoption, and innovation speed is described. Hence, to know what might enable or hinder employee innovation adoption, different barriers and enablers to innovation speed are addressed. Subject to barriers to innovation speed is the concept of defensive routines. This concept is explained from emotional tension and defensive reasoning/strategies on behalf of the employees within the hospital. This behavior is understood to slow down the pace of innovation adoption (barrier to innovation), hindering organizational innovation success. Hence, for innovation adoption, we argue that negative emotions (emotional tension) and defensive routines should not occur. This requires that trust and positive emotions must be present (see Fig. 1). Following the literature review comes an introduction to the hospital case, an explanation of the method used, and a combined “Results and discussion” section. Finally, practical implications, limitations and further research, policy implications, and a conclusion are made.

## Context

The Norwegian Work Life Model involves good working conditions between managers and employees where participation is a key factor (Ingvaldsen, Rolfsen, & Finsrud, 2012). The model contributes to a power balance between manager and employee, where co-determination for employees to plan and carry out their own working day ensures decision-making influence, involvement, and commitment. The model has thus resulted in a high level of trust between employees and management (Ingvaldsen et al., 2012). Innovation and efficiency are in this sense based on employees’ rights and opportunities to take responsibility. This contrasts with other work organization styles, e.g., scientific management, where competition and the ability to innovate were characterized by a focus on economic efficiency (Levin, 2012).

As there are complexities involved regarding employee motivation and managing improvement and progress in organizations, the following “Literature review” section seeks to highlight important aspects for employee innovation adoption.

## Literature review

In this section, a theoretical framework is provided to understand how trust may enhance innovation speed towards innovation adoption. The theoretical framework is structured as shown in Fig. 1. Figure 1 displays a *speed line* measuring *innovation speed* (pace). A high level of innovation speed leads to *innovation adoption*, whereas a low level results in a state of *standstill*. The process is as such a dichotomy and understood as continuous.

To achieve a high pace (innovation speed) of innovation adoption, we argue that there are mainly three factors that need to be considered: *emotion*, *defensive routines*, and *trust. Emotion* may be directed two ways (either towards standstill or innovation adoption) depending on different variables. *Defensive routines* will in this case only be directed one way as they are understood to reduce innovation speed. *Trust* may be directed both ways. The movement either from left to right on the speed line is thus dependent on these three factors. Consequently, for innovation adoption to occur, *emotion* should be at a positive level, *defensive routines* should be avoided, and *trust* needs to be present.

Trust and defensive routines are in this way discussed as two opposites towards innovation adoption: a higher level of trust reduces emotional tension, which reduces defensive routines and thus enhances innovation adoption pace. In effect, trust acts as a countermeasure (overrules) for emotional tension and defensive routines. In contrast, a lack of trust facilitates a sense of disconnect which may enable emotional tension and defensive routines towards the innovation, consequently reducing innovation adoption pace.

As a high level of innovation speed is understood to impact innovation adoption in this case, we acknowledge that a low or standstill level of innovation speed may be necessary in some instances for change to take place. Moreover, there exist difficulties with changing all variables impacting innovation adoption at once. For an elaborated version of Fig. 1 showing contributing variables to innovation speed as well as the connection between emotion, defensive routines, and trust, see Fig. 3.

## Theoretical framework to understand innovation speed

### Organizational innovation, innovation speed, and adoption

Organizational innovation is described as “a new or significantly improved knowledge management system implemented to better use or exchange information, knowledge, and skills within the firm” (Sapprasert & Clausen, 2012). Organizational innovation may be subject to the *adoption* of any type of novelty in an organization.

Innovation speed may be looked upon as “the time elapsed between (a) initial development, including the conception and definition of an innovation, and (b) ultimate commercialization, which is the introduction of a new product into the marketplace” (Kessler & Chakrabarti, 1996; Murmann, 1994). Hence, innovation speed involves the stimulating activities performed between initial ideas and the final product and is significant to create and sustain competitive advantage (Kessler & Chakrabarti, 1996). Innovation thus involves carrying *the occurrence* into practice (implemented or institutionalized) (Van de Ven, 1986). From an organizational point of view, innovation speed is associated with successful change by acquiring a *true sense of urgency* among a large enough group of people (avoiding negative emotions and complacent behavior) (Kotter, 2008). Innovation speed is as such dynamic and may vary according to various factors.

Decision involvement is argued to make it easier for commitment and acceptance (Vennix, Akkermans, & Rouwette, 1996), as well as facilitate a sense of dignity, community, and meaning (Weisbord, 1987). When introducing a new solution, Romme (2003) argues that involvement and participation should be done from the start for those who will carry out a new solution. Therefore, ignoring input from others (associated with traditional methods) can lead to a sense of uneasiness and a lack of trust (Stachowicz-Stanusch, Amann, & Mangia, 2017).

For the purpose of the hospital case, an emphasis is placed on organizational innovation (e.g., the new instruments and way of performing blood tests analysis), and the mechanisms in place (trust and defensive routines) which may enhance or hinder innovation adaption, adoption, and realization. Innovation speed in this case relates to the pace of innovation adoption which may impact the overall efficiency of the innovation implementation. Thereupon, organizational innovation relates to the new laboratory service situation, the degree of employee participation/involvement, and thus the pace of innovation adoption.

The next section will address some important barriers to innovation by looking at defensive responses from emotion and thus emotional tension.

### Emotional tension and defensive routines

Organizational changes might facilitate challenges regarding social structures and relationships (hindering innovation). Earlier studies addressing defensive routines in organizations (e.g., Whyte, 1949) on social structures of restaurants are important examples of how activity coordination is essential in connection with business growth. Emotional balance between employees may thus be provided from compensation; with an increase in one activity, one needs to decrease activity for the employee in other areas (Whyte, 1949). Furthermore, behavior from emotional tension is addressed in Donald’s (1959) study on a group of machine operators.

More recent views on defensive routines have been related to organizational theories of action, and how these theories may hinder or contribute to learning in organizations (Argyris & Schön, 1974; Argyris & Schön, 1996). Defensive routines from this view are described as “thoughts and actions used to protect individuals’, groups’, and organizations’ usual way of dealing with reality” (Argyris, 1985). It involves defensive reasoning and action strategies that seek to avoid embarrassment or threats (Argyris, 1991; Argyris, 2002). Defensive reasoning is about thought processes and cognitive rules that facilitate action (Argyris, 1991; Argyris, 2002).

Defensive routines have been described in various ways. For example, it may involve mixed messages (inconsistency) (Argyris, 1986), self-censorship (e.g., silence), and performing unilateral control through defensive reasoning approaches (Argyris, Putnam, & Smith, 1985). Defensive silence has been mentioned as deriving from fear of personal losses from speaking up (Dyne, Ang, & Botero, 2003). This is especially true for organizations where managers have given signs of not being interested in input from lower levels within the organization (Dyne et al., 2003; Hornstein, 1986). In the light of this, rational *self-interest-seeking behavior* is stated to derive in contexts where actors are *detached* from everyday routines (Bachmann & Zaheer, 2008). As separated activity/focus and lack of dialogue are associated with challenging social environments, facilitating dialogue and frictionless “cooler” environments may refocus group attention and attention towards the “living social processes that sustain them” (Bohm & Nichol, 1996; Fulmer & Keys, 1998).

Being accountable is mentioned as both an enabler and a barrier to organizational learning (Schillemans & Smulders, 2015). From this view, organizational learning and institutional accountability arrangements impact relationships (e.g., between an actor held to account and a forum holding the actor accountable). For instance, individuals tend to judge and make decisions based on accountability anticipation, e.g., *expectations* of having to justify feelings or beliefs to others (Lerner & Tetlock, 1999; Schillemans & Smulders, 2015). In effect, the threat of being accountable may enhance *self-criticism* and *defensive bolstering* (e.g., justifying positions to which one feels committed) (Schillemans & Smulders, 2015; Tetlock, Skitka, & Boettger, 1989). Nevertheless, conditions for learning relate to management structure (macro-level) and self-criticism from an actor’s anticipation of being accountable.

The decision to trust is understood to derive from reasoning (Argyris & Schön, 1996). As a result, defensive reasoning may hinder innovation speed. For this purpose, an emphasis is placed on employee experiences, and what may constitute defensive reasoning and strategies from an organizational perspective. To enhance the pace of innovation adoption by reducing defensive routines, the next section will introduce the concept of trust.

### Different perspectives on trust

Gambetta (1988) explains trust as “the probability that he will perform an action that is beneficial or at least not detrimental to us is high enough for us to consider engaging in some form of cooperation with him.” Bradach and Eccles (1989) describe it as a form of expectation that limits the risk of an exchange partner acting opportunistically. Similarly, trust is argued to guard against opportunistic behavior by “encouraging individuals to suspend judgment of others” (McEvily, Perrone, & Zaheer, 2003). It has thus been defined as mutual confidence that actors within an exchange will not exploit others’ vulnerabilities (Sabel, 1993). In this way, it is the perceived likelihood of another actor not operating in a self-centered manner (Madhok, 2006).

Trust is argued to influence “the density, multiplexity, stability, and non-redundancy of social structure.” In this view, *delayed reciprocity* is mentioned (McEvily et al., 2003). Delayed reciprocity and stability are about trust, explained to facilitate expectation of balance in future relationship exchanges (serial equity). This then minimizes the need for value or compensation coherence in single exchanges (enhances ability to manage uncertainty) (McEvily et al., 2003).

### Different dimensions of trust

Interpersonal trust is argued to involve two dimensions: cognitive and affective factors (Chae, 2016; McAllister, 1995). As cognition-based trust is about perceived expertise (confidence in others ability) and reliability of a partner (e.g., track record and reputation), affective-based trust involves emotional bonds (e.g., concern, caring, and faith in the trustworthy intentions of others) (Chua, Morris, & Mor, 2012). Hence, the type of trust provides different outcomes (e.g., variables) (Chua et al., 2012; McAllister, 1995).

Context is critical to understand trust, and various forms of trust may be mixed based on the situation. Therefore, conceptualizing trust in one form within a relationship is critical, as it may miss the rich diversity of trust in organizational settings (Rousseau, Sitkin, Burt, & Camerer, 1998). Recognizing that different relationships have various variations of trust, which may vary in terms of degree and setting, is thus important.

This paper seeks to provide a contextual description (case) of trust, discussing the implications of trust for innovation speed within organizations. In addition to a contextual definition of trust, the paper emphasizes an affect-based notion of trust between managers and employees. However, an emphasis is placed on the told experiences of hospital laboratory employees regarding the innovation situation. Thereupon, to understand the innovation situation as well as the role trust plays in innovation adoption, the hospital case will be introduced next.

## The case

### Case background

This case is based on a project (starting in 2015) involving the laboratory service and the implementation and centralization of new laboratory instruments for analyzing blood samples on behalf of a public hospital (enhance efficiency). The study is inspired by the hospital management’s wish for enhanced understanding of laboratory employee’s perceptions and needs in relation to facilitate innovation implementation success. The hospital operates in different geographical locations. This paper emphasizes four of these locations.

The hospital project report from 2018 states that work processes and organization should be developed in connection with increased automation and collaboration, both internally within the hospital laboratories and with the primary health service (clinics). All the laboratories related to one of the hospitals’ divisions were thus to have new analysis equipment adapted to various needs in place within the end of 2017. The project was divided into the following milestones:

- Project organization and project plans.
- Organization of a new workflow from patient needs, competence needs, and collaboration with the clinics.
- Acquisition of new analytical equipment.
- The implementation of new analytical equipment.
  *Due to, for example, complaint handling, the supplier contract was delayed and signed in March 2018. The project was decided to be completed after signing the contract, consequently transferring the responsibility for the equipment implementation, training of staff, method validation, routine operation, and disposal of old equipment to a new project subject to the operational organization*.

A project group was developed where one employee (subject coordinator) from each of the laboratory departments was represented. The subject coordinator from each group could thus contribute to decisions, efficient information flow, and coordination within the project. Furthermore, working groups (representatives/employees from each of the disciplines/geographies) would provide input with regard to requirements specification and choice of solution. Various dialogue meetings on behalf of the procurement and project information plans (e.g., status and orientations) were presented every half year at different locations. Additionally, project information plans (e.g., status and orientations) were presented every half year by the division director and/or project manager at different locations.

As part of the project with regard to the project distribution of blood samples from the primary health service, there were two models that were examined by the hospital division. The first model was related to the continuation of the current division of labor associated with separate laboratory analysis operations (current model). The second model consisted of collecting samples from the primary health service (associated with different geographical areas) and sending them to one of the hospital division laboratories (integrated model). The choice of model was based on an investigation of the organization in 2017 where an emphasis was placed on the consequences of the integrated model for service, quality, staff, and finances.

From the hospital division decision note (2017), the hospital division board concluded that a replacement of laboratory equipment would collectively represent an efficiency improvement that could be utilized in better quality, collaboration between laboratories, service, or financial savings. The alternative was to introduce a greater degree of automation of the sample flow. In this case, the investment need would be higher; however, with such a solution, it would be possible to achieve a more efficient operation. The report concluded that it would be most profitable to centralize most of the sample analyses to one location. Moreover, other analyses would be performed at the different hospital locations. However, analyzing samples from the internal hospital polyclinic would be done locally at each hospital division with new automated instruments. The procurement was carried out through a competition, where the supplier complied with various criteria and requirements specifications on behalf of the hospital. Hence, the innovation in this case is tailored to the hospital division needs, and thus related to the new way for employees to produce blood test analyses.

The new model distribution was proposed to provide the opportunity for professional specialization and establishing specialized expertise in the various areas. The centralization was mentioned as appropriate with regard to an optimal automated process from sampling to sample filing (reducing manual transfers and waiting time), in effect contributing to acceptable and predictable response times with regard to blood samples. By collecting, automating, and centralizing most of the analyses from the primary health service, it would enhance the capacity at the hospitals that no longer performed those analyses. The plan was thus to use this capacity for other quality and service-enhancing measures, hence strengthening the service initiatives towards polyclinic patients as well as the primary health service. At the same time, an emphasis would be placed at maintaining a good physical working environment, including training, service, and maintenance services.

The division director decided on a step-by-step development of the laboratory services through an integrated model that would form the basis for further organizational development and procurement. The project was mentioned to start with the replacement of equipment. Hence, a centralization and automation of tests from the primary health service would be initiated over a 2–3-year period.

### Challenges that emerged from the project

With regard to a workshop at the hospital in 2019, it was mentioned that the project was divided into two parts. Part 1 was completed and consisted of laboratory instruments/machines. Part 2 was the part that the hospital was facing (2019) and involved the organizational change/logistics. Nevertheless, the project was planned to be finished in May 2021.

Some challenges that emerged at the workshop based on the new model were related to competition, laboratory employees (e.g., emotions), and primary health care needs. In the light of this, the research has been aimed at understanding factors that contribute or hinder innovation adoption and thus efficient operation of the hospital’s laboratory service (sending, analyzing, and delivering blood samples to the primary health service). The laboratory service consists of the hospital divisions (subcontractor), primary health service (customer), and private laboratories (competitors). However, the main emphasis is placed on how the innovation impacts the hospital division’s (laboratory) employees and thus their experience with the present laboratory service. Therefore, mapping the needs on behalf of the hospital division’s employees was performed through in-depth interviews.

From the challenges that emerged, it is essential to understand what is really behind the respondents’ answers. The focus has thus been related to emotions, and how trust as a condition for innovation can affect the speed (e.g., pace of innovation adoption) of innovation. The role of trust between individuals for innovation, and what type of trust in this context contributes or hinders innovation adoption, has therefore been relevant. Consequently, by addressing barriers (e.g., defensive routines) to innovation on behalf of the hospital division employees, one can perhaps create an environment for innovation and change.

### The actors

Below is a description of the various actors relevant to the project. However, this case is limited to the interviews on behalf of the division’s employees.

#### The hospital and the hospital division (public operator/subcontractor/innovation holder/project owner)

The hospital consists of specialist health services. The hospital is organized with different divisions focusing on various health care areas. This case is thus based on one of these divisions (consisting of four laboratories placed in four different geographical locations) and their ongoing project.

#### “Quality assurance”

This company has a mission to improve the quality of the medical laboratory activities conducted. Therefore, it contributes to the other actors’ trust in that blood samples are analyzed and handled the right way before, during, and after analysis.

#### Primary health service (customer/partner)

The primary health service consists of the medical offices in the region that (to a greater or minor extent) uses the hospital’s laboratory services (e.g., transmission, analysis, and delivery of blood tests).

#### The competitor (private actor/subcontractor)

The case considers one of the hospital’s central competitors. This competitor was mentioned in the interviews with the hospital division employees.

An important difference between the hospital and their competitor is the fact that the hospital has two missions: taking care of patients at the polyclinic as well as handling the laboratory service towards the primary health service. However, their competitor only handles laboratory services. Hence, there is a difference in resource utilization and prioritization between these actors.

## Methods

For this paper, a qualitative investigation involving a case study and semi-structured interviews has been performed to understand how organizational innovational change impacts employee defensive routines and trust creation towards management.

The concept of trust has been argued to be *stretched* having a high level of abstraction and covering a broad dimension of meaning (Singh & Sirdeshmukh, 2000). Hence, changing the focus from *what is trust* to *which trust and when* has thus been argued to solve the confusion (Singh & Sirdeshmukh, 2000). Appropriate definitions of trust are argued to be highly context dependent (Goudge & Gilson, 2005). Hence, qualitative or experimental methods are common (e.g., semi-structured interviews, focus groups, or ethnographic approaches). These methods facilitate elaboration and a more detailed understanding of, for example, relationship experiences (Ozawa & Sripad, 2013).

By gaining insights on the experiences and needs of laboratory employees with the innovation at a specific point in time, important cues could be addressed to understand how trust may impact innovation adoption in this context. Moreover, the study facilitates insights which can make way for a more generalized quantitative study involving a larger health care network.

There was no relationship between researcher and participant prior to the interviews that could impact the study. A description of the research design and methods is explained as follows.

### Research design and method

To explore how trust may impact innovation speed, it has been essential to gain an in-depth understanding of the complexity of the laboratory service situation. Developing a contextual basis to describe and interpret emotions and their impact on innovation adoption has thus been important. In this sense, a case study approach has been used to develop a picture of the laboratory employees’ experiences with the innovation in their everyday setting (Yin, 2009). The case study approach is divided into three types: intrinsic (learning about a unique phenomenon), instrumental (gain a broader understanding of a phenomenon from a specific case), and collective (studying several cases at once) case studies (Stake, 1995). This study follows the description of an instrumental case study, as it involves gaining understanding of the context and impact of a realistic innovation implementation project on behalf of hospital employees. Moreover, as case study research emphasis on *how* and *why* questions, it is suitable for descriptive or exploratory studies (Myers, 2009; Ponelis, 2015). The study therefore seeks to address *how* employees have been affected by the innovation, *what* cues/mechanisms are contributing or hindering innovation speed and trust, as well as interpretations of possible reasons to *why* the mechanisms are important in this context. In this way, it acquires an interpretivist understanding of the meaning of employee experiences (Glaser & Strauss, 1968; Ponelis, 2015) within an organizational context.

Explorative and interpretive case studies usually develop descriptive frameworks and emphasis on the *number of cases*, *data collection techniques*, *unit of analysis*, *role of prior theory*, and *analysis methods* (Eisenhardt, 1989; Ponelis, 2015). To be able to assess the complexity of the laboratory situation, one case study has been chosen (Yin, 2009). In terms of *data collection techniques*, interviews are stated as the primary source of data for case studies (Yin, 2009). The data collection was divided into two phases that linked the contextual setting with employee experiences. Phase 1 involved acquiring knowledge about the hospital project (context and organizational structure), and to understand what factors were perceived as important for the innovation implementation. Hence, it involved workshop participation and meetings, as well as project documents (e.g., project reports). Phase 2 involved 1-h face-to-face interviews at the various laboratories which sought to gain in-depth insight into employees’ needs and perceptions, building on insights from phase 1. In relation to *the unit of analysis*, the in-depth interviews were performed with five key employees (women) from four different laboratories subject to the hospital division and the geographical area of study. The employees were chosen based on the division management’s suggestions (e.g., chosen from convenience and relevance to the study aim). However, the choice to have five participants was based on the complexity of the study, time considerations, and the value of gaining in-depth knowledge of employees’ experiences. As the interviews were recorded and transcribed in detail, ethical considerations involved communicating the promise of confidentiality and information (e.g., reason) about the interview as well as requesting informed consent from each respondent. Moreover, the interview transcription was sent by e-mail to which the respondents were free to depart from.

To be able to find various trust-creating mechanisms, the interviews were based on a semi-structured interview guide, created to facilitate a conversation surrounding the laboratory service network and relations. The Actors-Resources-Activities model (ARA model) (Håkansson & Snehota, 1995) with its emphasis on assessing the strength of actor bonds, resource ties, and activity links in organizational networks was thus chosen as a starting point and inspiration to develop interview questions. The questions provided an overview of the laboratory context as well as the important relationships, resources, and activities within them. Questions were related to important quality/value elements as well as missing work-related factors. Moreover, trust was stressed as an important component of actor bonds and an essential factor for enabling or hindering actor behavior in relation to each other (e.g., interaction) (Håkansson & Snehota, 1995). As the ARA model made it possible to understand the *bigger laboratory picture*, it was possible to narrow down the focus on understanding trust as a concept for innovation adoption within manager-employee relationships. To facilitate a basis for comparison between stated trust mechanisms on behalf of employees as well as trust mechanisms interpreted from the interview conversations, employees were asked one question directly related to what they thought as important trust-generating factors.

Phenomena within qualitative research are usually created from the meaning participants place on them (Daher, Carré, Jaramillo, Olivares, & Tomicic, 2017). In terms of *data analysis and interpretation*, there are various systematic procedures researchers may use. For example, an inductive approach starts with an area of study and allows theory to emerge from the data (Strauss & Corbin, 1990; Thomas, 2016). It involves summarizing raw data, creating relationships between research goals and raw data findings, and developing a theory or model about the visible structures or experiences present in the data (Thomas 2016). A deductive approach test if the data is consistent with earlier assumptions or theories identified or constructed. Moreover, many studies use both inductive and deductive approaches (Thomas, 2016); in this way, case studies support theory building (Yin, 2009) as well as theory testing (Eisenhardt, 1989). This study has taken inspiration from a combination of both approaches when analyzing the data, starting with an inductive approach involving a research question and the topic “trust.” The starting point was thus to understand what constitutes trust (trust mechanisms) on behalf of hospital employees in a specific organizational context (e.g., describing a picture of the phenomenon of trust being studied). Hence, *the role of prior theory* was subject to the analysis and interpretation (e.g., trust and defensive routines cues) part of the process as it was chosen after the interviews. An exploratory approach could in this way provide a descriptive framework (Rowley, 2002) as the intepretation part of the study started with only an assumption of various trust cues. Further, a deductive approach was conducted for the purpose of the discussion, and to be able to create implications. In this way, relevant literature was selected based on the inductive findings.

**Fig. 2:**
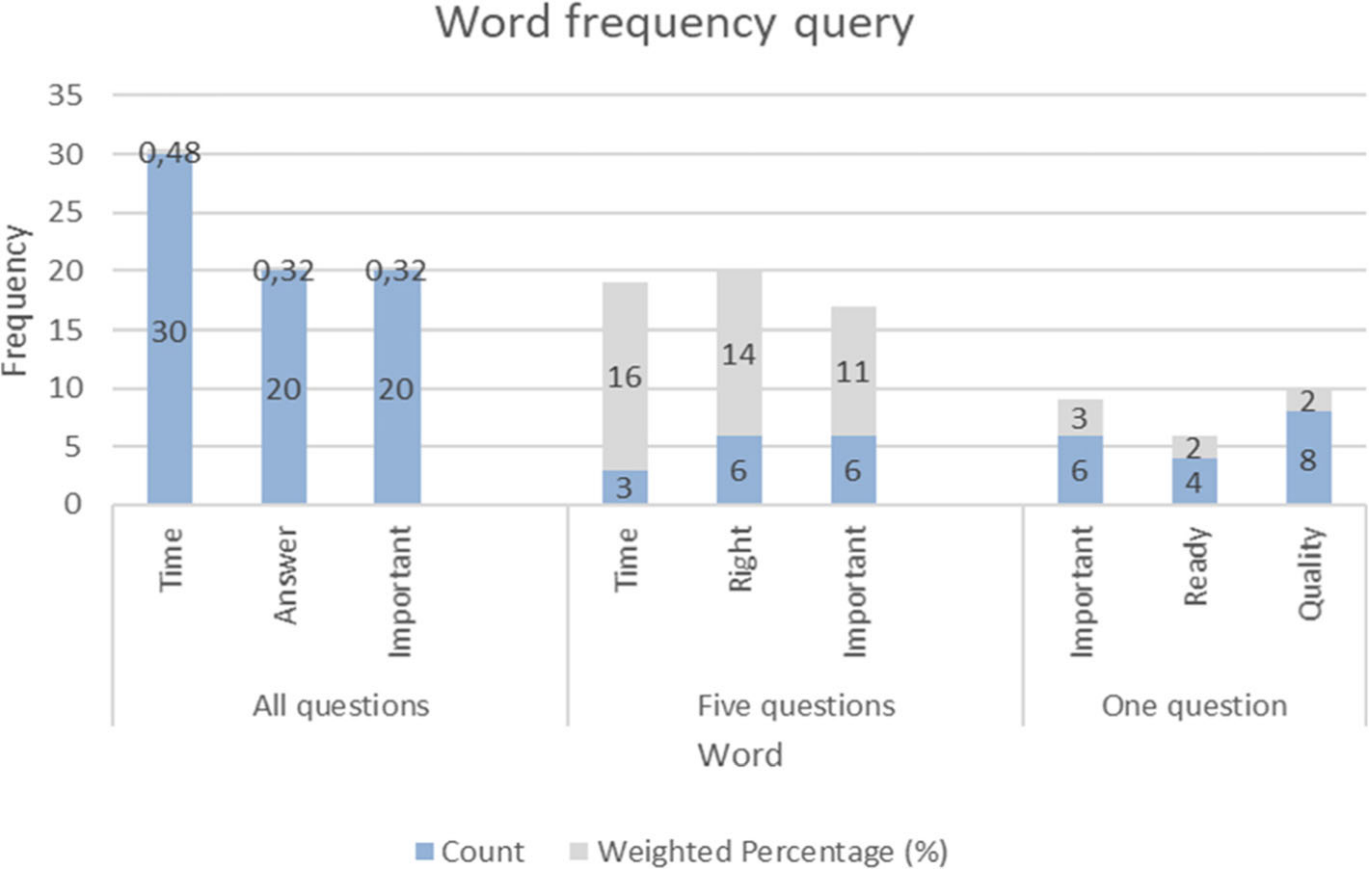
Word frequency query in NVivo. The Word frequency query displays the word count and the word’s weighted percentage

To develop a deep understanding of the specific case “seeking the phenomenological essences” (Bazeley, 2007), the inductive findings were based on an inductive coding process (Chandra & Shang, 2019). As such, the *analysis* of the interviews was performed in NVivo. Codes (in this case various mechanisms assumed as important for trust generation) were developed based on Word frequency query and Text search query, emphasizing on the words most frequently mentioned from the interviews and the words surrounding context (Fig. 2). Moreover, “coding is usually a mixture of data [summation] and data complication … breaking the data apart in analytically relevant ways in order to lead towards further questions about the data” (Coffey & Atkinson, 1996). As coding is stated as a cyclical act (Saldaña, 2016), providing an enhanced understanding of the data thus required an iterative process of recoding, as well as a dividing of the first code cycles into less and more refined codes. Moreover, to interpret the meaning of the codes to understand what mechanisms could impact trust generation, it was relevant to understand “what was going on” (Bazeley, 2007). How the respondent perceived the situation, what was happening, what they were trying to achieve, and how they were trying to achieve it (Emerson, Fretz, & Shaw, 1995; Saldaña, 2016) were thus relevant questions in terms of acquiring direction in terms making the codes. The inductive coding process (Chandra & Shang, 2019) thus made it possible to highlight important features of the data which facilitated the creation of various categories. These categories, when linked/compared with each other, simplified an understanding of patterns and connections within the data, which facilitated the development of the study’s themes and concepts (Bazeley, 2007).

### Validity

The analysis method the researcher uses to understand the respondents’ experienced reality has important implications for what results are communicated (Law, 2004). Regarding qualitative research, Kirk and Miller (1986) argue that validity is about “whether the researcher sees what he or she thinks he or she sees” in this way facilitating evidence within the data for interpretation. Hence, transparency and rigor are important elements (Tuval-Mashiach, 2017) and may be acquired from explicitly reporting how one accomplished what was achieved (Crawford, Leybourne, & Arnott, 2000). Providing a *detailed description* of the interview and analysis process has thus been essential. Furthermore, NVivo has been stated to add rigor to the analysis process (e.g., providing rapid and accurate searches, ruling out human error). Hence, validity regarding the results has been subject to the following (Elaine, 2002):

- The possibility of finding all instances of a specific usage (from large datasets).
- Combining manual and automatic processes for a thorough interrogation.
- The ability for rapid coding enhances confidence with data interpretation.
- NVivo makes an overview of what is going on easier, facilitating a seamless starting point for data analysis and interpretation. As researchers may interpret data differently, this enhances trustworthiness, rigor, and quality of the study.

The analysis process in NVivo has provided structure and confidence in the mechanisms developed. For this purpose, by performing three queries in NVivo (emphasizing different questions) (Fig. 2), it appeared that most of the factors under the question that was directly related to trust also emerged from the other words from the Word frequency query and Text search query. For this reason, it contributed to confidence and meaning regarding the trust mechanisms developed. Moreover, by using quotes from the interviews, the findings are grounded in the evidence.

According to Walsham (1993), validity of case estimation builds upon “the plausibility and cogency of the logical reasoning used in describing the results from the cases, and in drawing conclusions from them.” As the findings from this study derive from a single case study, it is context specific and provides in-depth insight, and the possibilities of generalizing the results are therefore limited.

**Fig. 3:**
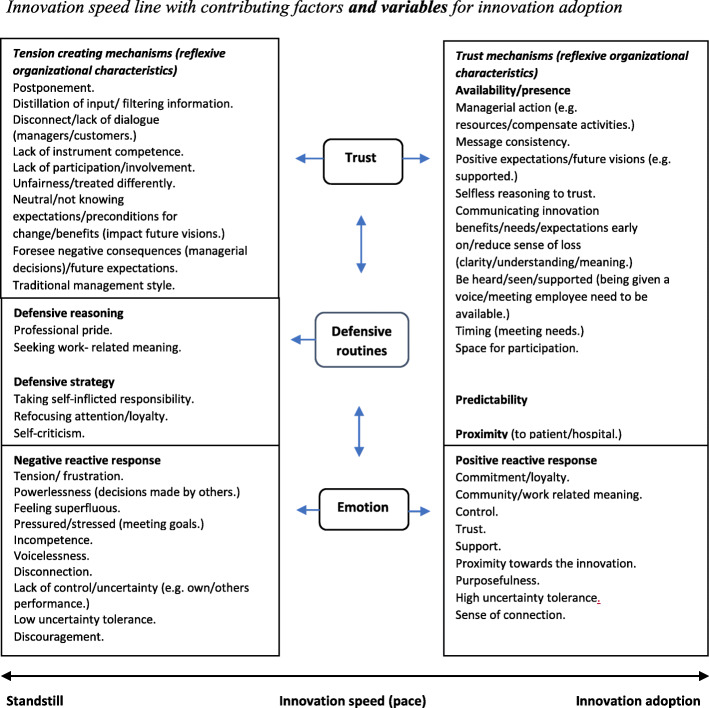
Innovation speed line with contributing factors and variables for innovation adoption

The two following “Results and discussion” section seek to highlight important findings (variables) and their importance for the innovation speed process (see Fig. 3) in the light of the theoretical framework (see Fig. 1).

## Results and discussion

To provide a contextual background that facilitates understanding for employee defensive reasoning and behavior (reactions) within the hospital case and the innovative environment (and how trust may address this), the innovation implementation and employee involvement situation will be discussed. The first part of the discussion explains the project environment. Hence, it involves insights made from given project reports and documents, as well as notes made from participating in a project workshop at the hospital in the fall of 2019. The second part of the discussion involves analysis of the in-depth interviews that followed.

### Background—framing the problem

To increase automation and collaboration internally and with the primary health service, the hospital division invested in new automated laboratory instruments in each of their laboratories. The organizational innovation in this case thus relates to employee experience with the implementation of the new way of performing laboratory analysis. The innovation is twofold and emphasizes a new way of working (automation/instruments/centralization) as well as innovation adoption of the new work situation.

The innovation implementation project resulted in a shift in laboratory equipment and work processes at the hospital laboratories. Employees at the hospital laboratories that were not part of the centralization thus performed other and fewer analyses. Hence, the new implementation situation had an impact on work routines and workload.

Various milestones were created for different purposes throughout the project. Relevant for this paper are the milestones involving implementation of new analytical equipment, which responsibility was transferred to another project. A main emphasis thus revolves around employees’ experiences with the implementation situation.

Positive implementation factors for the purpose of the laboratory employees related to dialogue meetings and the creation of project groups for the different laboratories to complete the project. Hence, one employee from each project group would act as a messenger between division management and the employees. In this matter, employees would be able to provide input regarding the project. From the hospital decision note (2017) (involving laboratory employee feedback), the choice of a new work model (instruments) was based on estimated consequences for service, quality, staff, and finances. However, consequences for employees related to more time to perform various routines. The step-by-step (2–3-year period) development of the laboratory service was stated to start with equipment replacement, followed by a centralization and automation of tests. However, the step-by-step introduction in addition to factors related to the board decision process had postponed the goal of having equipment in operation by 2017.

The first project milestone involved innovation implementation tasks related to acquisition of laboratory instruments and organizational development. However, from the project report, the organizational development part seems to have started with patient and primary health service needs, the skills needed to meet these needs, and appropriate work allocation and organization in the new workflow.

At the workshop, it was mentioned that the first part of the project involved implementing the instruments at the hospital laboratories. The second part of the project involving the organizational change (transportation and logistics of the samples) had just started with an estimated finish in spring 2021. From this insight, an assumption is made that the decision to implement the new instruments took place before considering employee’s needs. The instrument implementation was stated to ensure efficient and safe routine operation of all new equipment with good plans for training. Supplier training services on behalf of employees were thus stated to be included in the instrument procurement. However, it is unsure whether the training of employees had taken place before, during, or after the instrument implementation. As some employees stated a lack of instrument competence, that learning of the new instruments had been slow (see Table 2), and that some employees within the interviews had been busy the last years with training, it seems that the instrument training had not been optimal (not done before the implementation). In the light of this, the concept of *involvement* became relevant. As measures were performed to inform and include employees in the implementation (meetings, project groups, consequence measures), the possibility to participate seems to have involved giving inputs regarding an already decided implementation plan.

Several issues on behalf of the employees appeared at the workshop. For the purpose of this paper, three clusters were relevant: *personnel*, *employee emotions*, and *management*. From the employee’s utterances at the workshop, there seemed to be tension due to unresolved issues, uncertainties, and negative emotions regarding the new work situation. Input on behalf of the employees is stated in Table 2.

As a focus is placed on the employee experiences in this paper, some input points stressed by managers and employees on behalf of one of the cluster’s *management* have been gathered (see Table 1). Gathering points on behalf of both management and employees seeks to form a comprehensive picture of the hospital situation. In this sense, including insights on behalf of management seeks to provide perspective regarding the implications made. Moreover, the points served as an important starting point for the employee interviews that followed.

**Table 1:** Issues communicated at the workshop on behalf of the cluster “management”

Facilitating factors for employee response	
**Capacity pressure** (time/economy/instruments)	**Management**
	Part 1 of the project has not gone well. Too little capacity as all equipment was changed at once.
	Part 2 of the project is related to the success of part 1: “We should have been up and running the production in the spring of 2019, we are behind! How can we boost this timewise?”
	New automated instruments have not performed well. Part 2 is about collecting and transporting the blood samples (reducing transportation times).
	Management is pressured economically. Economically focused.
	Little time and capacity to be a leader (a lot of administrative work due to, for example, sick employees).
	Managers feel like organizers.
	Managers have many different tasks: adjusting what has been tough for the employees (project part 1), consider customer needs, enhance service (response times), and gather the laboratory to one community.
	Tiresome process for management with lacking resources and various project-related challenges (too many projects are connected to each other).
**Opportunities** Change for the better (strengthen bonds/relations)	Achieving closeness (bonds) to employees (hindered due to a lack of time).
	Get rid of negative emotions (help employees).
	Revitalize enthusiasm (towards entering a new project).
	Being a visionary (stated as important).
	The hospital needs help to address the workflow in each laboratory (transferring labor, job safety, and shifts need to match).

The facilitating factors from Table 1 indicate that management (like employees) was facing pressure regarding the innovation situation. As the project had taken longer time than anticipated, the situation seemed overwhelming. Moreover, the lack of resources (e.g., time) had placed pressure on managers to prioritize task which involved optimal operation of the new instruments and upholding service promises towards the primary health service (e.g., performing a *rematch* of the project part 1). In effect, the challenges from the first part of the project seem to have created more operational work in part 2 of the project. The problems in part 1 may thus be one reason for why management was lacking capacity to develop the relations with employees. Moreover, lack of coherence between laboratories (see Fig. 3) and gathering the laboratories to one community (see Table 1) were mentioned. Consequently, the complex organizational structure and installing various analysis instruments at different locations at once could have made dialogue and facilitating optimal learning of the new instruments more difficult.

Innovation adoption was argued to be socially deterministic, involving managerial action, human resources, and skills (Webb & Pettigrew, 1999). Moreover, not considering ideas from individuals of lower rungs may be a barrier to innovation (Yang & Konrad, 2011). As innovation in this sense was in relation to innovation creativity, not being open to employees’ needs may awaken innovation resistance from negative emotions. However, employees had strong opinions of the organization striving towards becoming a visionary (also stated in Table 2). As this was mentioned to relate to “striving to be the best in the world, not just small changes,” it may indicate a wish and motivation towards putting in the work of becoming a leading actor (if the right resources are in place). As negative emotions regarding the continuation of the project were stressed on behalf of employees, resources may relate to a larger extent of being able to participate and being heard with regard to the project (e.g., more dialogue and transparency). Moreover, stressing managers’ economic focus may mean a wish for closer relations (e.g., consensus with other actors within the organization) and being *seen* to a larger extent by management. Nevertheless, effects from part 1 of the project seem to have impacted part 2 negatively, changing work roles and workload on behalf of managers and employees alike.

**Table 2:** Issues communicated at the workshop on behalf of the cluster’s “personnel and employee emotions”

Employee response		
**Capacity pressure** (time/instruments/new routines)	**Personnel**	**Employee emotions**
	Employees do not have time to think about anything else but the new routines; employees are sick and do not have time to do the job they are intended to do. Employees are burned out from working overtime, and there is bitterness from the previous project part 1. Learning the new instruments have been slow (no use of virtual reality (VR) or augmented reality (AR) technology). As the new solution makes it possible to free resources, there is still a need to hire more expensive competence. A strong professional pride may be present. Employees need to adapt routines to their own workday. There are too many tasks for each employee. Employees have a locked mindset (e.g., what is in it for me?). One must consider the whole.	Employees are tired and unable to take risks in relation to continuation of the project (part 2) The project loyalty is weakened. Instruments do not work as expected. When instruments (automation line) do not work, this impacts employees’ professional pride/honor negatively. Feeling superfluous for lack of competence in relation to operating the new instruments (which are not working optimally) (e.g., wounded professional pride). Need to create motivation. “We must believe in the solutions that provide better service to hospitalized patients.” Resistance to changes/negative emotions. Negative emotions are difficult to get rid of (stated to be inherited between employees), e.g., rumors between employees of them not being allowed to perform certain tasks: “We are not allowed to do …” (The managers want to know how to get out of this in a stronger manner.)
**Opportunities**	Striving to be the best in the world, not just small changes. Being a visionary is missing. Get employees to see the opportunities in the project regarding safety delegates and employee representatives. “Is this enough? Where are the opportunities?” Willingness to change. How to make employees think differently?	Feelings of organizational measures being handled too late. Too much work pressure. This project (part 2) is an opportunity to operate differently.
**Hidden input**	Distillation of input; not everything seems to show (information on behalf of employees). Input from project meetings was *filtered* and in-depth arguments got lost.	

The managers seem to be aware of the various frustrations and wanted to empower employees towards project continuation (willingness to change). However, the pressured situation seems to be a barrier for this purpose.

Next, insights on behalf of the cluster’s *personnel* and *employee emotions* will be discussed (see Table 2).

Employee emotions from Table 2 indicate a lack of motivation and burnout from negative experiences and aftermath of the first part of the project (the term *burnout* was mentioned within the interviews). As the first part involved issues regarding learning and operating the instruments and the new routines that followed, it seems to have awakened negative emotions on behalf of the laboratory employees, which continued into part 2 of the project. This included bitterness (from part 1), reluctance to change, enhanced self-centeredness (e.g., “what is in it for me?”), and feeling superfluous in relation to poorly operating instruments and the lack of instrument competence (impacting professional pride and organizational loyalty).

As involvement and participation should be done from the start by those who decide on a new solution to facilitate commitment and acceptance (Romme, 2003), it seems that this has not been done in a timely manner. The decision to implement the new equipment and centralize some of the analysis to one location before considering employees’ (who directly work with the solution) needs from the start might thus hinder innovation speed. This is because not feeling included or being able to participate with the decision from the start may create a sense of reluctance towards the new solution. Negative rumors shared between employees may thus be the result of a disconnect (lack of dialogue) between management and employees which may keep the reluctance to change alive.

The sense of dignity, community, and meaning (Weisbord, 1987) was argued to be affected in this matter (impacting commitment and solution acceptance). The findings seem to complement this literature. In terms of dignity, the fact that employees felt superfluous by not having enough instrument competence (slow learning progress due to work overload) and having a reduced sense of professional pride in relation to the instruments not working as expected (not trusting the instruments), it may reduce innovation speed. The same is relevant from having a locked mindset (e.g., “what is in it for me?”), as it may reduce employees’ ability to feel a sense of community and meaning with the innovation. Similarly, some input on behalf of employees from previous meetings was stated as “filtered” such that some project-related arguments got lost. In effect, the organizational change phase (part 2 of the project) did not seem to firstly include employee’s needs. Hence, the sense of only being partly considered in the solution together with the feeling of input being “filtered” may in this case be one reason for the negative response towards participating in the second part of the project. Filtering employee feedback may thus be a barrier to innovation adoption as it excludes important information (e.g., needs/suggestions) on behalf of the employees, slowing down innovation progress. Barriers to innovation speed may in this sense be the result of (1) a combination of managers not having the capacity (due to a “rematch” of the project part 1) to consider employee’s needs and (2) employees not feeling heard. Hence, the stressful experiences from the project’s part 1 result in managers having to address various negative consequences in the project’s part 2. This postponement, together with a lack of employee participation due to prioritizing operational tasks (employees not feeling heard), may provide negative consequences for the pace of innovation speed. As it is unclear what has been filtered, not feeling heard may contribute to negative emotions and a lacking sense of meaning towards an efficient continuation of the project (innovation speed). Not feeling heard and feeling overlooked are therefore understood as contributing factors for negative responses (e.g., defensiveness) towards the continuation of the project (e.g., innovation speed).

Management clearly states a wish to empower their employees. For this reason, this paper looks at how trust may rebuild and turn defensive responses towards a willingness to continue the project (e.g., positive responses) in relation to the innovation situation. In this sense, the insights from the first part of the paper (e.g., workshop and various project documents) have given relevant knowledge on issues which frame the laboratory service context (see Table 1). Moreover, the issues are understood as contextual factors which might facilitate defensive responses and thus behavior towards the innovation.

To gain a deeper understanding of employee’s experiences with the new laboratory service situation, in-depth interviews were performed with key laboratory employees at each of the four laboratories. The next section involves these conversations and the assumingly defensive behaviors that derived from the told experiences (interviews). The three words most frequently mentioned from all the interviews were *time*, *answer*, and *important*. Additionally, the words *importan*t and *time* appeared in two of the other analyses. Therefore, an extra emphasis is placed on these words and their meaning. By performing these analyses, it was possible to focus the interview content to answer the research question and create trust mechanisms. The results from NVivo are presented in Fig. 2.

The trust mechanisms are understood to be essential factors that impact employee trust generation towards management and the innovation (see Fig. 3). Moreover, as part of the various trust mechanisms, an assumption of facilitating factors for defensive behavior is created and is understood to impact trust in this context. The discussion is based on the trust mechanisms, as well as facilitating factors that are understood to place barriers for trust generation (e.g., contribute to defensive behavior) (see Fig. 3). As defensive behavior is believed to reduce innovation speed in this paper, the insights provide a basis for how trust may impact innovation speed from defensiveness. To answer how trust may impact innovation speed, the next sections will address defensive routines and trust from the in-depth interviews.

### Defensive routines

Defensive routines are argued to involve reasoning (e.g., thoughts and cognitive rules) and action strategies which seek as protection to avoid embarrassment, pain, or threats (Argyris, 1991; Argyris, 2002). For the purpose of this paper, an emphasis is placed on defensive routines (defensive strategies and reasoning) from what is told within the interviews. However, as defensive reasoning involves mental processes, only an assumption could be made of employees’ defensive reasoning. What is described as facilitating factors for defensive routines is thus understood as the responses from the interviews (involving emotion) which may impact defensive reasoning and strategies, consequently impacting trust generation and innovation speed negatively.

Bachmann and Zaheer (2008) mention self-interest-seeking behavior resulting from detachment from routines. However, self-centered reasoning may in this case result from the combination of not feeling heard/overlooked by management (disconnect/lack of dialogue between managers and employees), as well as upholding professional pride. This is due to a lack of competence and/or the sense of being superfluous regarding operating instruments, which have resulted in a lack of loyalty towards the continuation of the project (see Fig. 3).

Emotional tension may rise in organizations where a compensation for new activities is not provided (Whyte, 1949). In this sense, activity coordination was stressed as important in times of business growth. For this purpose, as employees were feeling burned out due to the changes in routines, it seems that there is a need to compensate activities to regain emotional balance. As negative rumors were present and stressed to be *inherited* between employees (see Table 2), the sense of *not being allowed* to do certain activities might have contributed to transferring tension between employees and units (Whyte, 1949), collectively “slowing down” (e.g., hindering) innovation speed.

From the in-depth interviews, negative responses portraying tension regarding the new situation resulted in one noticeable (key) defensive strategy: *taking responsibility*. Moreover, this strategy contained various subcategories of defensive routines (e.g., defensive strategies and reasoning). As the interview results are categorized into what is assumed as mechanisms impacting trust creation, an explanation of the defensive routines will be performed for each trust mechanism (availability, predictability, proximity, and one question of trust) (see Fig. 3). In this matter, taking responsibility firstly involved self-interest-seeking behavior (Bachmann & Zaheer, 2008), and separated activity/group attention (Bohm & Nichol, 1996; Fulmer & Keys, 1998). The lack of dialogue with management thus seems to impact employees’ *attention towards something/someone else* (e.g., the primary health service), *professional pride*, and *seeking meaning*. Moreover, the sense of feeling responsible facilitated *self-criticism* (Schillemans & Smulders, 2015; Tetlock et al., 1989). The four subcategories of defensive routines subject to *responsibility* will be discussed and addressed with relevant trust literature as follows.

### Focusing attention as a result of responsibility

As no additional resources had been added regarding the organizational change, the employees who had extra tasks did not have time to do this, nor inform the primary health service regarding routine errors. Employees were therefore afraid that bad habits would be formed.

“I have worked overtime to be able to order items and have them available, so it’s a very unbearable situation. There are limits to how much you can handle. And then we have always said how important it is that we act on these things (…) that we have an updated laboratory handbook, that we hold courses, get to travel and inform and that we are active in relation to these things.”

Some employees did not feel heard or prioritized. The answer indicates that employees may have felt discouraged and pressured to reach analysis goals, as management had waited to handle the challenges they were facing. At the same time, new knowledge needed to be acquired on behalf of handling the new instruments and routines.

Employees who were not directly involved with the new instruments did not feel prioritized. Hence, employees might have felt frustration and a lack of control (uncertainty) from not feeling supported in relation to the new situation. Moreover, it may be the sense of not being able to be sufficiently *available* towards the primary health service. Therefore, it had raised concern (emotional tension) towards management and the innovation (disconnect/detachment from management), consequently resulting in self-interest-seeking reasoning in terms of enhanced responsibility (defensive strategy) towards the primary health service. Employees were thus directing focus away from the innovation efforts (e.g., redirecting attention and loyalty) from self-interest and disconnect with management, and the innovation.

### Professional pride and seeking meaning as a precondition for responsibility

The innovation situation led some employees to be afraid of not being able to use their education and what they were trained for. In this way, employees seemed to perform self-protection regarding work titles by demonstrating clear boundaries of what their job really was all about.

“We are [profession] to analyze blood tests, which is why we have chosen this profession. It’s something about maintaining an interesting position for everyone so we don’t lose staff or get in trouble with the recruitment.”

Employees felt a great deal of uncertainty about an unclear situation where some of the premises for the change and cooperation were not known. In this sense, *redirecting* loyalty towards the primary health service seemed to be a defensive strategy by taking control of the situation from *responsibility*. Hence, with a lack of managerial support and task direction, employees were protecting professional pride (and the sense of feeling superfluous) from creating work-related meaning. Redirecting attention in this way may thus be a result of tension from not feeling heard by management. Therefore, taking responsibility seems to be the result of seeking meaning (professional pride) and gaining control of the unclear situation. Hence, in this case, self-interest-seeking behavior may be described as self-interest-seeking reasoning. This is because it involves thought processes which seem to somewhat justify and manage the overwhelming situation by creating meaning. This type of reasoning may guide (come before) responsible behavior (defensive strategies) (see Fig. 3). Consequently, as defensive routines are described to hinder learning in organizations (Argyris & Schön, 1974; Argyris & Schön, 1996), it may hinder innovation speed by redirecting attention (e.g., loyalty) from self-interest-seeking reasoning.

Being a member of “Quality assurance” was stated to provide assurance (e.g., predictability) in that routines would be performed in the right manner. However, uncertainty towards own performance and not being in the position to make decisions seemed to impact employees’ sense of pride in being portrayed as a skilled employee. As a result, the employees became more aware of their own strengths and weaknesses. Hence, they attempted to communicate their strengths by identifying factors that distinguished them from their competitors, namely *proximity* to the hospital and the patient. One employee pointed out a personal and passionate cause over the last 15 years for maintaining test samples (especially when it was cold outside) during transportation. Employees therefore took responsibility and were loyal towards their customers by defending their position from justifying strengths. Justifying weaknesses from strengths in the context of responsibly may therefore be a type of defensive reasoning. Adhering to and taking responsibility for personal causes, despite a lack of compliance, may thus provide evidence for employees’ need to make sense of the situation, mean something, and be seen. In this way, the fact that employees participated in regular meetings without feeling heard (e.g., from the sense of information being filtered) may indicate a sense of voicelessness (involuntary silence). Innovation speed and thus organizational capability may in this case be reduced from voicelessness and a lack of participation.

### Self-criticism as a result of responsibility

As a result of the innovation and the new routines, the hospital division’s laboratories had a strong wish for change, in relation to being given more time to provide better laboratory service towards meeting primary health service’s needs (wished this was perceived as an important task). In this case, some employees were self-critical (blaming themselves) for feeling *responsible* for the lack of presence. One employee took the blame (self-criticism) for not listening properly and not understanding the primary health service needs.

*“…and then there is the doctor’s office visits that are far too rare. That is because I do not allocate my time properly.”*

The employees knew that the hospital had enough resources. However, the fact that they did not feel prioritized (without understanding why) may have provided frustration due to the sense of being treated differently (e.g., unfairly).

*“I want us to change to be able to provide more services, but some issues are placed at a level that we have no control over. Then there is no use.”*

Nevertheless, the current regional solutions were considered to be an impediment for being present.

Being accountable was stressed as both positive and negative for learning (Schillemans & Smulders, 2015). However, as the employees in this case did not seem to be accountable for the lack of dialogue with the primary health service, they might have felt responsible due to the pressured situation. In this way, it may be possible that employees were taking responsibility due to not knowing managers’ expectations as well as the uncertainty towards own performance (lack of dialogue/disconnect towards management). Not knowing the preconditions for change, uncertain environments and tension may frame anticipations of management and/or the innovation which limit positive expectations (e.g., Clegg et al., 2002) with regard to the innovation (e.g., needs not being met). As not knowing might make it harder to create expectations of what might happen in the future, it seems that this uncertainty had impacted actors to enhance defensive routines. The responsible strategy may act as a defensive mechanism to protect (e.g., a sense of risk reduction from believing that the experienced behavior would continue into the next part of the project) and gain control regarding the unclear situation. Hence, a defensive strategy from anticipations may be *self-inflicted responsibility* in relation to *neutral anticipations* from uncertainty and disconnect towards the innovation/management. In this sense, justifying weaknesses from strengths in the context of responsibly might be a form of defensive bolstering. Nevertheless, as responsibility (defensive strategy) is positive towards tasks related to the primary health service, it does not contribute to innovation speed (e.g., redirecting attention).

The next section will discuss how variations of trust may impact innovation speed, by reducing defensive routines.

### Increasing innovation speed from trust

For the purpose of trust, this concept was stated to vary depending on degree and setting. Understanding *what type of trust* is present is therefore relevant. To overcome defensive routines and facilitating change, acquiring an awareness of the mechanisms driving trust and tension on behalf of the employees has been important to know how innovation speed may be increased.

In this case, trust seeks to increase innovation speed (adoption). As redirecting attention and loyalty (an outcome of taking self-inflicted responsibility) is understood to be a defensive strategy that reduces innovation speed, finding the right trust mechanism that reduces emotional tension and the sense of disconnect, enhances work-related meaning, and focuses attention on the innovation is important. What is described as trust mechanisms (see Fig. 3) are from the interviews and analysis understood to be important factors that impact employees’ experiences and thus emotions (e.g., tension) towards the laboratory service. For this reason, as trust initiatives (e.g., trust mechanisms) are understood to impact emotions and defensive routines and thus the ability to trust, there exists a connection between the three variables *trust*, *emotion*, and *defensive routines* (see Fig. 3). However, it is important to keep in mind the complex multi-location laboratory structure (e.g., lack of coherence) and the challenges with the instruments, which in this case seems to have placed barriers for the management and employee dialogue and connection.

How the various trust and tension-creating mechanisms may impact trust, and reverse defensive routines in this context, will be explained next.

Trust was stated to be associated with expectations of being heard, of positive responses, or from receiving innovation benefits (Clegg et al., 2002). Furthermore, it was stressed to link to the probability of beneficial actions (Gambetta, 1988). Not being able to be sufficiently available towards the primary health service and not feeling heard, prioritized, or been given enough resources (compensate activities) to perform all the needed tasks are therefore understood as tension-creating mechanisms. These have thus enhanced uncertainty towards the innovation and the way management has handled the situation. Redirecting attention and loyalty away from the innovation might in this sense be impacted from employees being able to foresee negative consequences of management decisions. As employees might feel they are in a better position (proximity to the primary health service/competence) to know what is best for their customers, not being considered may place a barrier to trust generation.

As the tension had been physically experienced by the employees over time (e.g., burnout), discouragement had been formed from not feeling heard (e.g., experienced negative responses from management). The combination of having communicated needs, and the sense of important issues being filtered and addressed at a later point, may thus have framed future expectations towards management in a way that had limited the belief that the innovation was beneficial (disconnect) (benefits are less likely to happen). This belief may thus have contributed to employees finding their own ways by taking responsibility (defensive strategy/action) from self-interest, e.g., professional pride (defensive reasoning). This is to reduce tension in terms of directing attention towards what is perceived as important (proximity to the primary health service), and which provide benefits (in this case work-related meaning, e.g., professional pride/feeling superfluous/competent/personal causes and situational control). Attention and loyalty, which are perceived as conditions for trust generation, are in this way directed towards the primary health service, by making sure they were doing things the right way (responsibility as a defensive strategy for self-protection) (Probst & Büchel, 1997). For this reason, innovation speed may be enhanced from trust by communicating innovation benefits towards employees from the start (e.g., Romme, 2003) of the innovation implementation. This is because enhanced clarity/performance certainty, innovation understanding, and training as well as feeling heard may limit employees’ need to cope, hold on to what is familiar/manageable (e.g., previous routines), and having to justify and compensate for their experienced and assumed weaknesses. However, innovation speed is only assumed to be enhanced if mixed messages (Argyris, 1986) are avoided in this case. This is because tension was created by not having experienced the told benefits (e.g., being given more time for favored tasks) during the project part 1. As being given more time was one of the original ideas with the innovation (communicated in meetings), challenges and the uncertainty with part 1 of the project had made this benefit difficult to comply. Consequently, addressing this issue at a later point in time had triggered defensive responses regarding the innovation situation. Time therefore seems to be an important dimension in this case in terms of tension creation, and a factor which may impact when a message becomes *mixed* and when defensive reasoning starts. Knowing this boundary is meaningful for message consistency/predictability, which is understood as significant for trust and innovation speed in this case.

Creating a space for employee participation where employees feel heard is understood as essential to reduce negative rumors and self-interest-seeking reasoning and tension. In this way, trust generation is understood to start when tension-creating mechanisms are reversed (taking action) by management (see Fig. 3). The amount of tension-creating mechanisms addressed might thus state something about the level of trust generated between management and employees, impacting the probability for innovation adoption. As defensive reasoning is connected to defensive strategies/action, reversing tension-creating mechanisms may impact selfless reasoning to trust (e.g., overruling defensive self-interest-seeking reasoning) due to positive expectations of management facilitating innovation benefits. Hence, defensive reasoning may be looked upon as part of the process to trust management and the innovation. In this way, trust may be perceived as an outcome of employees’ selfless reasoning, due to the act of reducing emotional tension (tension-creation mechanisms), disconnect, and defensive reasoning towards management and the innovation. In this way, the defensive strategy of responsibility may, from trust, be redirected back towards the innovation (alter the sense of *proximity* towards the innovation), consequently increasing innovation speed.

By feeling heard, supported, and gaining the needed resources to be available, it may enhance employees’ beliefs of being supported in the future (e.g., delayed reciprocity) (McEvily et al., 2003). Moreover, expectations of support, clarity, and meaning with the new situation may provide a sense of acceptability and uncertainty tolerance (McEvily et al., 2003). As *predictability* was understood to be important for the employees, employees may be guided to trust by expectations of being heard/supported (reasoning to trust). Consequently, trust might enhance the tolerance for the laboratory situation being uncertain, directing attention and loyalty (e.g., acceptance) towards the innovation. *Speeding up* might in this sense involve reduced tension and enhanced sense of connection (dialogue) with management, limiting defensive routines. Moreover, self-criticism is assumed to link to uncertainty towards own and others’ performance, and a lack of control (e.g., feeling powerless and frustrated) due to a lack of resources given to perform optimally regarding the innovation. As the employees wished the tasks towards the primary health service were looked upon as important (being given resources), expectations of being supported in this matter seemed to be limited. Being self-critical could therefore be the result of taking responsibility from uncertainty tolerance being low. As being accountable enhanced self-criticism (Schillemans & Smulders, 2015; Tetlock et al., 1989), the fact that employees took responsibility (self-inflicted responsibility) on such a high level when they were not expected to shows the value of communicating expectations and needs for innovation speed (facilitating positive attitudes, e.g., selfless reasoning) towards management intentions with the innovation. Managerial action thus frames expectations and willingness to adopt the innovation.

It is important to keep in mind that finding the right balance for trust depends on various factors (e.g., change in organizational structure, management availability, and needs). In this case, the laboratory structure (organizing style) as well as the pressured situation for management (see Table 1) seems to have created distance between managers and employees. Moreover, the fact that the hospital was mentioned to be governed by others (e.g., government level) and various agreements plays an important part in relation to managers’ ability to perform acts of trust. As the tension-creating mechanisms are assumed as essential for trust generation in this case, they might vary in importance and change between employees at different points in time. Moreover, as reducing tension-creating mechanisms may make the situation more bearable for the employees, it does not mean that the goal of innovation adoption is reached.

The paper findings indicate that innovation implementation decisions have been made without sufficient consultation and regard of the employees’ knowledge and experience.

A more traditional approach to management and change seems in this way to have impacted employees negatively. Consequently, the organization style in this case seems not to be consistent with the traditional Norwegian Work Life Model. Furthermore, we argue that trust is an important factor to enhance innovation speed. However, as trust creation is highly complicated, it is hard to break it down and analyze it. As a result, trust in this case may be understood as a consequence of positive emotions employees may develop based on *organizational characteristics* (e.g., management decisions, atmosphere, communication/dialogue, and participation/involvement). From this view, trust is understood as *reflexive*, modified from a reactive response to the experienced organization style.

### Practical implications for innovation speed

Enhancing technology (medical instruments and equipment) is essential to increase blood analysis efficiency and in this way meet patient needs in better ways. For urgent and critical hospital situations (e.g., the COVID-19 situation), we argue that speed is an important element for innovation implementation success. Moreover, as urgent situations often involve making fast decisions, technical knowledge, achieving common objectives, and professional responsibility place a special emphasis on the importance of the ability to trust management.

Successful innovation implementation in organizations requires managers that take action towards enhancing the connection with their employees. As this case has shown, negative rumors, self-interest-seeking reasoning, and tension are factors which might reduce innovation speed. Creating a social environment by facilitating a space for employee participation where employees feel heard and supported (e.g., empowered) is therefore essential. This involves providing positive responses to employee’s needs (tension-creating mechanisms), which may impact innovation understanding and frame employees’ positive expectations of the innovation being beneficial.

Reducing the sense of loss and focusing attention on the innovation can be done by providing meaning and protecting employees’ professional pride. Therefore, managers should provide enough information for the reasons and consequences for innovation implementation (information regarding resources, competence, being able to use education). Being available for the employees as well as facilitating the needed resources for employees to feel available (e.g., proximity) towards the primary health service may thus produce positive emotions and a sense of predictability. This might impact future expectations of being supported (from positive reasoning to trust), consequently limiting employees from performing defensive routines.

As trustful actions by management are assumed to link to positive expectations from selfless reasoning, facilitating resources (compensating activities, avoiding postponing problems, and taking action) may limit employees’ sense of uncertainty and lack of control (towards own competence, the context, and customer needs). This might reduce self-inflicted responsibility and self-criticism, shifting the focus towards the innovation. In the light of this, facilitating transparency and dialogue of expectations and needs towards communicative tasks involving the primary health service might reduce the disconnect between managers and employees. In effect, reducing the sense of having to manage tasks and take responsibility alone (self-inflicted responsibility) may impact positive expectations of managers’ decision-making abilities.

### Limitations and further research

We are aware that there are other views that may provide different perspectives to the study.

For the purpose of innovation adoption, this could involve alternative approaches to scientific management, e.g., employee-driven innovation or workplace innovation. Moreover, as speed could be a function of a sense of urgency (e.g., COVID-19) (Kotter, 2008), the concept of trust subject to the importance of speed for urgency, and having a shared vision, could be a topic for further research in relation to different organizations facing rapid change. In this sense, a focus could be placed on corporate transformation (facilitated by a shared vision of the intended outcomes of the transformation). Moreover, issues of autonomy, participation, and forms of participatory action research could be explored to take the case forward. Equally important, the ability to trust might change depending on context (e.g., organizational structure or availability of management). In this sense, one might investigate the relevance of time as a dimension for tension-creation and defensive routines in this context.

As the study describes a context-specific description of trust in one specific situation, the implications made to generate trust may vary in other settings. Generalizing trust and tension-creating mechanisms for innovation speed within the health sector thus means that more studies on this issue are needed. In the light of this, we acknowledge that the ability to trust is complex and based on various factors. As we recognize a connection between level of trust and defensive routines, this connection needs further research. For example, degree of defensive reasoning and routines, and the ability to trust may, in addition to management and organization style, vary depending on deeper human characteristics (e.g., psychology, sociology, anthropology) placed outside of the boundary of this paper. Thereupon, by going deeper into each individual employee need, one might reveal new mechanisms, which could be employee specific, to increase innovation speed (individual level). In this case, it could be possible to provide enhanced insight regarding the mechanisms driving defensive reasoning (e.g., professional pride) on behalf of each individual. This could facilitate learning in relation to motivation measures for selfless reasoning facilitating a linking of individual and organizational levels for innovation speed. Similarly, as we observe a connection between *emotion*, *defensive routines*, and *trust* (see Fig. 3), a better understanding of the appropriate levels (e.g., amount/balance of variables subject to the three factors) that must be present for innovation adoption to occur (turning point) in this context is needed.

In relation to emotional tension, e.g., *stress* and *burnout*, we acknowledge some of the complexities of using these terms to the context of hospitals, as there exist different understandings of the terms among disciplines. Additionally, 3 months is not considered enough time to fully understand the complexities of the overall situation. Hence, we highlight the importance of stress and burnout as terms having various connotations among disciplines. Therefore, to seek a more accurate explanation of what stress and burnout mean in this case, the facilitating factors for defensive routines/tension-creating mechanisms (see Fig. 3) are a description of what social and environmental factors (that over time) might have contributed to employees’ response. Additionally, the amounts of tension-creating mechanisms might impact the level of trust generated between management and employees. However, as only an assumption could be made of the link between level of trust and probability for innovation adoption, investigating this connection in relation to defensive routines could be valuable.

As interviews were performed on behalf of employees, creating a context including management has involved workshop notes and reports. Information, reports, or measures taken place beyond this point in time have thus not been included in the study. As the project report does not state anything more than organizational development being postponed to another project, only an assumption could be made on this part being addressed in the project’s part 2 from information at the workshop.

Moreover, since this paper has taken the employee perspective in a complex organizational structure, further research could involve defensive routines on behalf of management. This might provide in-depth insight of the “why” of defensive routines developed in this case. Moreover, it would give more input on relational and dynamic connections regarding defensive routines and how they might vary and change between organizational levels. Furthermore, the concept of self-interest-seeking behavior (e.g., professional pride and control) and thus meaning creation from tension may connect to the concept of “sensemaking” (e.g., Weick, Sutcliffe, & Obstfeld, 2005) and could be a form of “negative sensemaking” which may link to trust generation. This connection is worth investigating.

### Policy implications

Being able to take part in a politically and regionally governed public innovation system (e.g., regional hospital structure) has made it possible to yield important insights for decision-makers and future policy decisions within the context of innovation and structural change (e.g., innovation centralization). The study results have contributed to lifting the discussion with regard to the regional innovation system by providing a glimpse into the effects of a structural hospital change associated with a lack of employee involvement. We therefore argue for the importance for policy makers to consider employee (e.g., innovation users) voice and participation (starting from the initial stages of the decision-making process) to avoid the development of defensive reasoning and routines as it may slow down the innovation adoption process. Understanding what cues breed a higher level of commitment and trust towards management and the innovation may in this way boost innovation progress. The findings lay forward political guidelines to important incentive systems politicians and hospital division managers can initiate to enhance the pace of innovation adoption in a structural change context. In this way, the study has facilitated a framework with significant factors the authorities may use for innovation understanding. Moreover, understanding the importance of addressing the darker side of innovation is significant for patients and the society (e.g., urgent situations and crisis). Accordingly, it may simplify the process of earning financial support for research, innovation, and sustainable growth (e.g., The Norwegian Research Council or Innovation Norway).

## Conclusion

To help organizations with innovation implementation success, a focus has been placed on important mechanisms driving trust creation for innovation speed towards innovation adoption in the context of the Norwegian Work Life Model. By investigating hospital employees’ experiences with implementing new laboratory instruments for blood test analysis, tension-creating mechanisms understood as barriers to innovation speed could be addressed.

The study shows that employee emotional tension within a context of organizational innovation and complex organizational structures facilitates disconnection and defensive routines towards management and the innovation. This involves self-interest-seeking reasoning (e.g., professional pride) and defensive acts of self-inflicted responsibility, which may redirect employee’s attention away from the innovation efforts and towards what is perceived as meaningful. Consequently, the study provides a new and contextual understanding of defensive reasoning and behavior for trust and innovation speed. To enhance innovation speed from trust, the study discusses relevant types of trust mechanisms applicable for this case, emphasizing on the importance of managers’ role in creating a space for employee voice and meaning. Timing, availability, communicating expectations, participation, and addressing various emotional tension-creating mechanisms are in this sense understood as essential elements which may impact positive reasoning to trust. Having a human-centered focus throughout the innovation implementation process is thus understood as equally important to enhance trust and the pace of innovation adoption, as the innovation itself.

## Data Availability

The datasets used and/or analyzed during the current study are available from the corresponding author on reasonable request.

## Abbreviations

ARA model: Actors-Resources-Activities model
NVivo: Qualitative data analysis software
VR: Virtual reality
AR: Augmented reality
COVID-19: Coronavirus disease 2019
